# The Association between Methionine Intake and Diabetes in Chinese Adults—Results from the China Health and Nutrition Survey

**DOI:** 10.3390/nu15010116

**Published:** 2022-12-27

**Authors:** Xiaomin Sun, Yingxin Chen, Jing Shu, Zhongying Li, Dongmei Yu, Wen Peng, Alice F. Yan, Youfa Wang, Zumin Shi

**Affiliations:** 1 Global Health Institute, School of Public Health, Xi’an Jiaotong University Health Science Center, Xi’an 710061, China; 2 Key Laboratory of Trace Element Nutrition of National Health Commission, National Institute for Nutrition and Health, Chinese Center for Disease Control and Prevention, Beijing 100050, China; 3 Nutrition and Health Promotion Center, Department of Public Health, Medical College, Qinghai University, Xining 810061, China; 4 Division of Research Patient Care Services, Stanford Health Care, Palo Alto, CA 94305, USA; 5 Human Nutrition Department, College of Health Sciences, QU Health, Qatar University, Doha 2713, Qatar

**Keywords:** animal methionine, plant methionine, diabetes, urbanization, interactive effect

## Abstract

This study aimed to evaluate the association between methionine intake and diabetes prevalence in Chinese adults and explore whether the association was source-specific. Data from 12,849 adults aged ≥20 years old were used from the China Health and Nutrition Survey during 1997–2011. Diabetes was diagnosed as self-reported and/or when blood tests results met the diagnostic criteria. A 3-day, 24-h recall was used to assess different sources of methionine. Multivariable mixed linear regression was used to examine the associations. Across the quartiles of total methionine intake, the odds ratio (ORs, 95% CI) of diabetes were 1.00, 1.49 (1.21 to 1.82), 1.72 (1.37 to 2.15), and 2.53 (1.97 to 3.23). In the subgroup analysis, similar trends were observed in both animal and plant methionine. There was a significant interaction between urbanization and diabetes. The positive association was only significant in those who lived in low or medium urbanization areas. The ORs (95% CI) were 1.00, 1.27 (0.85 to 1.88), 1.56 (1.01 to 2.39), and 1.79 (1.09 to 2.95) for medium urbanization, respectively. No interaction was identified when stratified by different methionine sources. In conclusion, methionine intake was positively associated with diabetes independent of food source, and it was modified by urbanization levels.

## 1. Introduction

Non-communicable chronic diseases (NCDs) have become the leading cause of mortality and disease burden worldwide, while diabetes is one of the most common NCDs globally [1]. Estimates from the International Diabetes Federation (IDF) suggest that 537 million people had diabetes in 2021, and the number is projected to reach 783 million by 2045 [2]. The most recent nationally representative surveys among Chinese adults reveal that from 2013 to 2018 diabetes prevalence increased by 13.8% [3]. Individual lifestyle behaviors (such as diet and being overweight/obese) and macroeconomic factors (e.g., income levels, urbanization characteristics) have been consistently linked to the risk of diabetes [4,5,6].

Long-term dietary patterns and compositional interventions have beneficial effects on the prevention and treatment of diabetes [7]. Methionine is notably the first amino acid present in nuclear encoded proteins, as the methionine codon signals the start of protein translation. Recently, evidence suggests that methionine restriction (MR) is positively associated with an increased life span and metabolic health through multiple mechanisms, including increased fibroblast growth factors (FGFs) and reduced fasting insulin and blood glucose [8,9,10]. Using glucose tolerance tests, Malloy and colleagues found that the mean insulin response to the oral glucose load was significantly less in the MR group than their counterparts, with comparable blood glucose between groups indicating that MR was the differentiating factor in this significant reduction [11]. Consistently, documented studies of both rats and mice indicate that MR reduces fasting insulin and improves glucose tolerance overall or in specific tissues (e.g., liver, muscle, and adipose tissues) [9,12,13,14]. Viewed together, these findings consistently indicate that a lower methionine intake plays a vital role in the prevention of diabetes.

Despite the clear benefits of methionine in laboratory animal models, evidence from the human population focused on the association between methionine intake and diabetes, was rare. One 16-week dietary intervention with MR (>80% relative to controls) in subjects with obesity and a metabolic syndrome reported that MR did not alter insulin or glucose directly, but enhanced the shift from carbohydrate to fat oxidation, which was closely involved in glucose metabolism [15]. On the other hand, the same nutrients from different food sources may show different effects on glucose metabolism [16]. A prospective cohort reported that after a mean follow-up of 16.9 years, adults with the highest quartile of methionine intake (Q5) had a 2.5 times greater risk of diabetes-caused death than Q1, while the associations became not significant when animal and plant-based protein was further added in the adjustment, indicating that this relationship may be largely influenced by different foods [17].

Compared with plant-based sources, animal food sources generally have higher amounts of methionine, although the proportion varies substantially [18]. Along with rapid urbanization and modernization, more methionine provided by animal foods (such as meat, fish, poultry, eggs, and dairy products) is now entering the human food supply than at any time in history [19]. However, to our knowledge, no studies have investigated the influences of source-specific methionine intake on the incidence of diabetes using longitudinal data analysis. Such information is of significant importance for identifying targets for T2DM management using nutrition interventions.

Using the longitudinal survey data collected in the China Health and Nutrition Survey (CHNS) during 1997–2011, our aim was to assess the long-term association between total methionine intake and diabetes among adults and determine whether the association was source-specific.

## 2. Materials and Methods

### 2.1. The Study Design and Study Sample

This study is an association analysis based on repeated measurements of data on dietary intake and diabetes using the CHNS data. The CHNS is an ongoing open-cohort study and profound cooperation project between the China Centers for Disease Control and Prevention (CCDC) and the Carolina Population Center at the University of North Carolina at Chapel Hill. Since its establishment in 1989, CHNS has been used to collect 11 panels of data in 1989, 1991, 1993, 1997, 2000, 2004, 2006, 2009, 2011, 2015, and 2018 using a multistage random-cluster sampling method. The composition of the CHNS includes more than 30,000 participants from 15 provinces in China [20]. Blood samples were only collected in the 2009 and 2015 surveys, whereas the latter was not open to the public.

Individuals with extreme values of energy intake (men: <800 kcal/day or >6000 kcal/day and women: <600 kcal/day or >4000 kcal/day) were excluded [21]. The analyses were limited to those who completed at least two nutrition surveys and had self-reported and/or objective diabetes diagnosed from data during 1997–2011. Finally, a total of 12,849 participants aged ≥20 years who met the above criteria were included in the present study. The mean follow-up was 9.0 years (standard deviation 4.5) during 1997–2011. In total, 27.1%, 16.0%, 17.8%, 19.0%, and 20.1% of the participants had 2, 3, 4, 5, and 6 times of measured nutritional evaluation, respectively.

### 2.2. Outcome Variable: Diabetes

The primary outcome was diabetes. Self-reported diabetes was identified based on the questionnaire if an individual answered “yes” to the question “Have you ever been told by a doctor that you have diabetes?” Additionally, a fasting blood sample was obtained in 2009 with diabetes defined as a fasting glucose of ≥7.0 mmol/L or the hemoglobin A1c (HbA1c) of ≥48 mmol/mol (equivalent to 6.5%) [22]. Thus, the prevalence of diabetes in 2009 was ascertained if a participant had self-reported diabetes or if their blood tests results met the diagnostic criteria. Fasting plasma glucose was measured with the glucose oxidase–phenol aminophenazone method (Randox Laboratories Ltd., Crumlin, UK). The HbA1c was measured with an automated glycohemoglobin analyzer. All measurements and tests were performed by trained professionals using standard protocols. The data collection protocol is described in detail elsewhere [20].

### 2.3. Exposure Variable: Dietary Intake of Methionine

Individual dietary intake data were collected by trained investigators who conducted a 24-h dietary recall on each of 3 consecutive days at each wave using a standardized questionnaire. Food purchased from markets, harvested from gardens, and foods and condiments in the home inventory were weighed and recorded by interviewers at the beginning and end of the 3-day survey period. The types and amounts of foods, the types of meals, and the places of consumption for each participant were tracked from both dietary recall and the records kept by the individual. Cooking oil and condiment consumption for everyone in the household was estimated using an individual energy-weighted intake. A detailed description of dietary measurements has been published previously [23]. In addition, the dietary assessment method has been validated [24].

For this study, the food intake data from 1997–2011 were recorded and converted to nutrient intake. We used the 3-day average food intake data to calculate the intake of nutrients, including total, animal, and plant methionine, according to the Chinese Food Composition Table [25]. The cumulative mean intake of total animal and plant methionine for each individual in each period was calculated to reduce intra-individual variability and represent long-term habitual intake [26]. For example, if a participant attended the survey in 1997, 2000, and 2004 at the age of 23, 26, and 30 years with an intake of x, y, and z, then the cumulative mean intake was calculated as (x + y + z)/3.

### 2.4. Covariates

Socioeconomic and lifestyle factors were evaluated using a structured questionnaire. The socioeconomic status (SES) included: annual family income (recorded as low, medium, and high), education (low: illiterate/primary school; medium: junior middle school; high: high middle school or higher), and urbanization levels (recorded as low, medium, and high). The lifestyle factors included: (1) the level of physical activity (the metabolic equivalent of task), which was calculated based on self-reported activities and duration using a Compendium of Physical Activities; (2) smoking status, which was categorized as non-smokers, ex-smokers, and current smokers; and (3) alcohol consumption, which was recorded as yes or no.

The BMI (body mass index) was calculated as weight (kg) divided by height square (m^2^). According to the Chinese standard, being overweight and obesity were defined as having a BMI of ≥24.0 kg/m^2^ [27]. Hypertension was defined as a systolic blood pressure ≥ 140 mmHg and/or a diastolic blood pressure ≥ 90 mmHg, using antihypertensive treatment or reporting “yes” when asked if the participant had known hypertension.

### 2.5. Statistical Analyses

The mean intake of cumulative methionine was recorded as quartiles. Descriptive statistics were calculated using the mean ± standard deviation (SD) for continuous variables and *n* (%) for categorical variables. The characteristics at baseline according to the quartiles of total methionine intake were compared using a one-way analysis of variance (ANOVA) for continuous variables and chi-squared tests for categorical variables.

The association between methionine intake and diabetes was assessed using mixed effect logistic regression models. Unadjusted and adjusted odds ratios (95% confidence interval (CI)) of the fixed part of the models were reported. Adjusted models were built by including age, gender, and energy intake initially in Model 1; adding education, income, urbanization, smoking, alcohol drinking, and physical activity in Model 2; and then adjusting for dietary patterns, BMI, and hypertension in Model 3. In the subgroup analyses, the multiplicative interaction between the different sources (animal or plant-based) of methionine intake and the covariates (sex, age, education, income, urbanization, smoking, overweight, and hypertension) was tested by adding a product term into the regression model. In the fully adjusted model, we also conducted a restricted cubic spline with three knots at 10, 50, and 90th to assess the non-linear association between methionine intake and diabetes. We found no evidence of a non-linear association.

The analyses were performed using STATA 17.0 (Stata Corporation, College Station, TX, USA). A *p* < 0.05 was considered statistically significant (two-sided).

## 3. Results

### 3.1. Descriptive Results

Table 1 shows the sample characteristics of 12,849 Chinese adults aged ≥20 years old divided by quartiles of methionine intake. The mean age was 43.3 years old (SD 14.8) and 49.0% were men. Across the quartiles (Q) of methionine intake, the mean ± SD methionine intake was 894.7 ± 152.6, 1236.8 ± 81.8, 1540.2 ± 100.3, and 2156.3 ± 455.8 mg/d. The cumulative animal methionine intake increased from 187.3 ± 177.6 in Q1 to 1075.9 ± 568.6 mg/d in Q4, and plant methionine intake showed a slight increase from 707.3 ±191.6 in Q1 to 1080.4 ± 433.6 mg/d in Q4. Furthermore, across Q1–Q4, the total energy, fat, protein, carbohydrates, fresh fruit, vegetables, and meat intake showed increasing trends. Adults with higher incomes and education levels or those with higher urbanization levels had a higher methionine intake. The total prevalence of hypertension and diabetes were 15.9% and 2.1%, respectively, and the lowest prevalence was observed in Q3.

### 3.2. Associations between Total, Animal, and Plant Methionine Intake and Diabetes

Mixed-effect models showed the associations between total, animal, and plant-based methionine intake and diabetes in adults aged ≥ 20 years (Table 2). Across the quartiles of total methionine intake, the odds ratios (ORs, 95% CI) for the prevalence of diabetes were 1.00, 1.49 (1.21 to 1.82), 1.72 (1.37 to 2.15), and 2.53 (1.97 to 3.23). Although they were weakened after further adjustment, the positive associations were still observed in models 2 and 3. When stratified by specific source of methionine, similar trends were observed in both animal methionine and plant methionine. Across quartiles, the ORs (95% CI) were 1.00, 0.98 (0.76, 1.26), 1.33 (1.01, 1.75), and 1.52 (1.13, 2.04) for the animal-based methionine intake, and 1.00, 1.12 (0.91–1.38), 1.26 (0.99–1.60), and 1.52 (1.14–2.02) for the plant-based methionine intake in model 2; the positive associations were attenuated and became only marginally significant after further adjusting for dietary patterns, BMI, and hypertension status.

### 3.3. Subgroup Analyses of the Associations between Quartiles of Different Methionine Intakes and Diabetes

**Figure 1:**
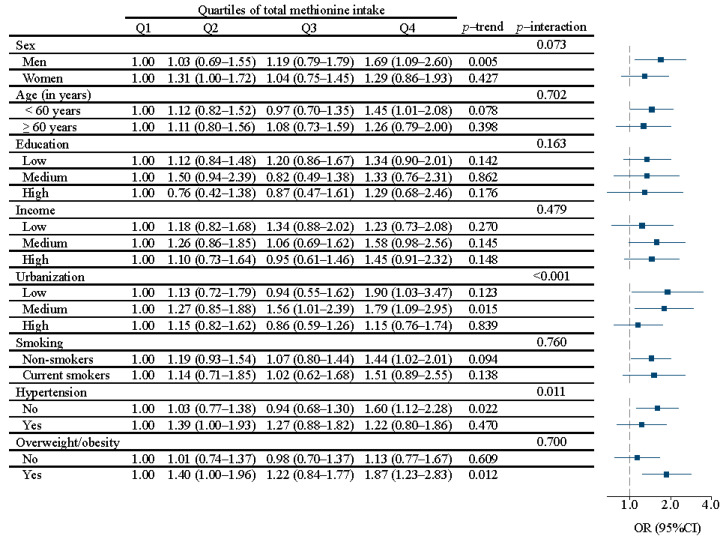
Subgroup analyses of the association between quartiles of total methionine intake and diabetes. Values are odds ratios with a 95% CI from mixed-effect logistic regression models. The model was adjusted for age, gender, energy intake, education, income, urbanization, smoking, alcohol drinking, and physical activity, dietary patterns, BMI, and hypertension. Q, quartile. Stratification variables were not adjusted in the corresponding models. A forest plot showing the associations between methionine intake in Q4 and diabetes in China.

Interestingly, there was a significant interaction between urbanization (*p* < 0.001) and hypertension status (*p* = 0.011) in relation to diabetes (Figure 1). Across Q1–Q4, the positive associations were only significant in those who lived in low or medium urbanization areas and those without hypertension. The ORs (95% CI) were 1.00, 1.27(0.85–1.88), 1.56 (1.01–2.39), and 1.79 (1.09 to 2.95) for medium urbanization, and 1.00, 1.03 (0.77–1.38), 0.94 (0.68–1.30), and 1.60 (1.12–2.28) for those without hypertension, respectively. No interaction was identified when stratified by animal or plant methionine (Table 3 and Table 4).

## 4. Discussion

Among the 12,849 participants who completed at least two nutrition surveys in the CHNS, across the quartiles of methionine intake, the intake derived from an animal source increased by 4.7 times, while plant-based intakes only rose by 52.7% from Q1 to Q4, respectively. Meanwhile, those with a higher methionine intake were more likely to have diabetes independent of the food source. In addition, the positive associations between total methionine intake and diabetes were stronger in adults with lower urbanization levels or who did not have hypertension. To our knowledge, this is the first longitudinal study that has reported such associations in Chinese adults.

Since the opening up of China, the economy of the country has constantly and rapidly increased, which may greatly affect the Chinese diet and related malnutrition issues. Data from the nationally representative study showed that the dietary structure of Chinese adults has changed significantly, characterized as a reduced intake of cereals and vegetables and increased intake of meat, eggs, fish, and dairy [28]. This shift in nutritional intake may contribute to the differences observed across the quartiles of methionine intake in our study: animal-based methionine intake increased by 4.7 times from Q1 to Q4, while plant methionine intake increased by only about 50%.

Compelling evidence from animal models shows that a lower methionine intake could reduce the incidence of diabetes [9,11,12,13,14], while the functional relevance in the general population remains to be elucidated. Two previous intervention studies reported that a lower methionine intake had no effect on insulin and glucose levels, although some other biomarkers (e.g., FGF-21) related to insulin sensitivity were improved. These two interventional trials had small samples with short interventional terms (a 16-week or 7-day term), and subjects were obese and/or reported as having a metabolic syndrome, which may limit the effect of MR intervention in the regulation of their diabetes [15,29]. Inconsistent with the literature, we found a positive association between total methionine intake and diabetes, independent of dietary pattern, BMI, or hypertension. Additionally, the associations were greater in adults with a lower urbanization level and without hypertension. In general, adults with lower urbanization levels were less likely to get better nutrition and meet nutritional recommendations. Supportively, the CHNS, using 2004–2011 data, reported that urbanization appears to positively affect rural residents’ healthy food preferences and dietary knowledge, which plays an important role in the prevention of diabetes [30]. Our results also showed that across the quartiles of methionine intake, the intakes of meat and fat increased by 4.4 times and 89.6%, respectively, compared with only a slight increase in fresh fruit and vegetables.

On the other hand, we failed to observe a significant interaction between methionine intake and education and income levels in relation to diabetes. Generally, individuals with higher education or income levels were more likely to have healthier food habits and meet nutritional recommendations [31], which may reduce diabetes’ risk. However, the association was inconsistent. Recently, findings from 29 nationally representative surveys indicated that diabetes prevalence was increased with higher educational and income levels in low- and middle-income countries, which was completely different from the results in the high income countries [32]. Due to the rapid economic development in China, the disparity in education and income has expanded significantly during the study period [33]. This may attribute to the negative association in our study. Furthermore, adults with hypertension were mostly taking antihypertensive medication, which may alter the association between methionine intake and diabetes. Finally, despite being overweight or obese having a close association with diabetes, we failed to obtain a significant interaction with methionine intake in relation to diabetes. This may suggest that mechanisms other than being overweight and obese are responsible for the association between methionine intake and diabetes. More investigations are warranted to elucidate the association between methionine and diabetes.

This study also found that the same nutrients from different food sources may show different effects on health. A recent review documented that a higher intake of plant protein was associated with a lower risk of diabetes, while the association was divergent for animal protein [16]. Another prospective cohort consistently reported that the positive association between methionine intake and the incidence of diabetes-caused death was largely affected by animal and plant protein [18]. Although this study proposed that the associations would be altered by different methionine sources, a positive association was identified in both plant and animal methionine intake in the present study. Notably, a high ratio of plant-based food in Chinese food culture usually represents a relatively high intake of heavy metals such as lead [34]. The positive association between plant methionine intake and diabetes may be due to increased levels of heavy metals (such as lead), which may contribute to a higher diabetes’ risk [35].

Our study has several strengths. First, we used the CHNS database to select a prospective, large sample of a nationally representative cohort, to make repeated measurements of dietary intake and diabetes. Second, the data were originally obtained through a 3-day 24-h diet record recall, which increased the reliability and validity of long-term methionine intake. Third, in addition to the large sample size, the data were drawn from different cities and rural areas in different provinces in the country, which make our results highly promotable. Fourth, several known and potentially confounding factors were adjusted in our analyses. Our study expanded on previous evidence and is the first to support the positive link between different methionine intakes and diabetes in the Chinese population.

This study has several limitations.. First, the ascertainment of diabetes was self-reported except for 2009, which might pose a misclassification of the outcome. However, the associations between food and nutrient intake with diabetes were validated by other studies using data from the CHNS [21,36]. Although we controlled our analyses for many factors to minimize confounding, we could not exclude the possibility of residual confounding. Second, this study did not distinguish between type 1 and type 2 diabetes, although the association would be unlikely to change significantly. Chinese population-based data indicated that the type 1 diabetes onset peak was in the 10–14-year-old age group [37]. In the present study, among the 1219 participants who self-reported having diabetes, only 30 cases (2.5%) were under 20 years of age. Third, the association between methionine intake and diabetes seems not to be altered by age group (stratified by 60 years). Fourth, although dietary pattern, a confounding factor, was fully considered in the present study, the associations of methionine intake within different dietary patterns are important to explore. Fifth, although the CHNS used a well-validated self-reported 3-day dietary intake survey to quantify dietary nutrient consumption, it may reflect the long-term intake. Finally, we were unable to explore potential physiological mechanisms due to a lack of related methionine biomarkers.

In conclusion, methionine intake was positively associated with risk of diabetes among Chinese adults independent of different methionine sources. There was a significant interaction between methionine intake and urbanization levels, showing the increased risk of diabetes in those with higher total methionine and lower urbanization levels. Further research is needed to elucidate the association between methionine and diabetes at the population level.

## Figures and Tables

**Table 1:** Sample characteristics of Chinese adults aged ≥20 years old who completed at least two nutrition surveys in the China Health and Nutrition Survey during 1997–2011 by quartiles of methionine intake (*n* = 12,849).

	Q1	Q2	Q3	Q4	*p*
	*n* = 3213	*n* = 3212	*n* = 3212	*n* = 3212	
**Age, mean (years)**	46.5 (16.3)	43.7 (14.5)	41.8 (14.1)	41.3 (13.6)	<0.001
**Sex**					<0.001
Men	1082 (33.7%)	1413 (44.0%)	1725 (53.7%)	2069 (64.4%)	
Women	2131 (66.3%)	1799 (56.0%)	1487 (46.3%)	1143 (35.6%)	
**Survey year**					<0.001
1997	1786 (55.6%)	1961 (61.1%)	1878 (58.5%)	1608 (50.1%)	
2000	579 (18.0%)	494 (15.4%)	490 (15.3%)	513 (16.0%)	
2004	420 (13.1%)	335 (10.4%)	357 (11.1%)	470 (14.6%)	
2006	194 (6.0%)	161 (5.0%)	170 (5.3%)	209 (6.5%)	
2009	234 (7.3%)	261 (8.1%)	317 (9.9%)	412 (12.8%)	
**Socioeconomic factors**					
Education					<0.001
Low	1669 (57.5%)	1420 (48.7%)	1200 (41.0%)	959 (32.4%)	
Medium	790 (27.2%)	913 (31.3%)	996 (34.1%)	1042 (35.2%)	
High	443 (15.3%)	580 (19.9%)	728 (24.9%)	962 (32.5%)	
Urbanization					<0.001
Low	1425 (44.4%)	1148 (35.7%)	932 (29.0%)	783 (24.4%)	
Medium	874 (27.2%)	1017 (31.7%)	999 (31.1%)	895 (27.9%)	
High	914 (28.4%)	1047 (32.6%)	1281 (39.9%)	1534 (47.8%)	
**Lifestyle factors**					
Smoking					<0.001
Non smoker	2427 (75.8%)	2243 (69.9%)	2068 (64.6%)	1890 (58.9%)	
Ex-smokers	52 (1.6%)	39 (1.2%)	52 (1.6%)	49 (1.5%)	
Current smokers	723 (22.6%)	927 (28.9%)	1081 (33.8%)	1271 (39.6%)	
Alcohol drinking	778 (24.7%)	1014 (32.2%)	1216 (38.5%)	1466 (46.6%)	<0.001
Physical activity, mean (MET-hrs/week)	134.2 (117.2)	143.0 (116.3)	145.2 (117.5)	139.6 (116.7)	0.001
**Weight status**					
BMI (kg/m^2^), mean (SD)	22.5 (3.3)	22.7 (3.3)	22.6 (3.1)	23.0 (3.2)	<0.001
BMI ≥ 24 (kg/m^2^)	847 (29.0%)	909 (30.9%)	895 (30.1%)	1041 (35.0%)	<0.001
**Dietary intakes**					
Energy intake (kcal/d)	1757.8 (433.6)	2152.9 (469.2)	2405.7 (522.2)	2735.5 (677.0)	<0.001
Fat intake (g/d)	48.0 (27.2)	60.8 (29.8)	71.0 (32.0)	91.0 (41.1)	<0.001
Protein intake (g/d)	46.9 (10.7)	61.7 (11.5)	73.6 (13.9)	92.4 (22.4)	<0.001
Carbohydrate intake (g/d)	282.1 (87.4)	336.4 (102.5)	363.6 (119.2)	378.4 (139.6)	<0.001
Cumulative methionine intake (mg/d)	894.7 (152.6)	1236.8 (81.8)	1540.2 (100.3)	2156.3 (455.8)	<0.001
Cumulative animal methionine intake (mg/d)	187.3 (177.6)	368.3 (238.8)	578.9 (302.6)	1075.9 (568.6)	<0.001
Cumulative plant methionine intake (mg/d)	707.3 (191.6)	868.5 (234.3)	961.4 (294.6)	1080.4 (433.6)	<0.001
Methionine intake (mg/d)	894.7 (152.6)	1236.8 (81.8)	1540.2 (100.3)	2156.3 (455.8)	<0.001
Animal methionine intake (mg/d)	187.3 (177.6)	368.3 (238.8)	578.9 (302.6)	1075.9 (568.6)	<0.001
Plant methionine intake (mg/d)	707.3 (191.6)	868.5 (234.3)	961.4 (294.6)	1080.4 (433.6)	<0.001
Intake of fruit (g/d)	14.9 (53.5)	18.6 (85.6)	25.8 (80.8)	35.3 (94.6)	<0.001
Intake of fresh vegetable (g/d)	235.7 (148.9)	267.1 (156.0)	291.0 (172.7)	326.9 (204.7)	<0.001
Intake of meat (g/d)	28.7 (35.7)	57.7 (52.7)	88.5 (69.7)	153.9 (119.8)	<0.001
**Disease history**					
Hypertension	517 (17.5%)	466 (15.7%)	417 (13.9%)	493 (16.5%)	0.002
Diabetes	57 (1.8%)	54 (1.7%)	49 (1.5%)	106 (3.3%)	<0.001

Data are presented as mean (SD) for continuous measures, and *n* (%) for categorical measures.

**Table 2:** The association between total, animal, and plant methionine intake and diabetes among adults attending the China Health and Nutrition Survey.

	Q1	Q2	Q3	Q4	*p* _trend_
**Total methionine**					
Model 1	1.00	1.49 (1.21–1.82)	1.72 (1.37–2.15)	2.53 (1.97–3.23)	<0.001
Model 2	1.00	1.32 (1.06–1.66)	1.27 (0.99–1.63)	1.84 (1.40–2.43)	<0.001
Model 3	1.00	1.19 (0.95–1.49)	1.09 (0.85–1.40)	1.49 (1.12–1.98)	0.009
**Animal methionine**					
Model 1	1.00	1.27 (1.02–1.60)	2.19 (1.75–2.75)	2.68 (2.11–3.41)	<0.001
Model 2	1.00	0.98 (0.76–1.26)	1.33 (1.01–1.75)	1.52 (1.13–2.04)	<0.001
Model 3	1.00	0.93 (0.72–1.21)	1.26 (0.95–1.67)	1.37 (0.99–1.89)	0.014
**Plant methionine**					
Model 1	1.00	1.02 (0.84–1.23)	0.92 (0.74–1.15)	0.83 (0.64–1.06)	0.107
Model 2	1.00	1.12 (0.91–1.38)	1.26 (0.99–1.60)	1.52 (1.14–2.02)	0.004
Model 3	1.00	0.99 (0.81–1.22)	1.11 (0.88–1.41)	1.28 (0.96–1.70)	0.079

Values are odds ratios with a 95% CI from mixed-effect logistic regression models. Q, quartile. Model 1 was adjusted for age, gender, and energy intake. Model 2 was further adjusted for education, income, urbanization, smoking, alcohol drinking, and physical activity. Model 3 was further adjusted for dietary patterns, BMI, and hypertension.

**Table 3:** Subgroup analyses of the association between quartiles of animal methionine intake and diabetes.

	Q1	Q2	Q3	Q4	*p* _trend_	*p* _interaction_
**Age (in years)**						0.896
<60 years	1.00	1.09 (0.78–1.53)	1.44 (1.00–2.06)	1.56 (1.04–2.35)	0.015	
≥60 years	1.00	0.78 (0.52–1.17)	1.00 (0.64–1.56)	1.16 (0.69–1.96)	0.331	
**Sex**						0.087
Men	1.00	0.71 (0.47–1.07)	1.07 (0.70–1.66)	1.28 (0.79–2.05)	0.056	
Women	1.00	1.13 (0.81–1.57)	1.46 (1.00–2.12)	1.42 (0.91–2.23)	0.070	
**Education**						0.809
Low	1.00	0.82 (0.60–1.12)	0.98 (0.68–1.41)	1.07 (0.69–1.66)	0.622	
Medium	1.00	1.28 (0.73–2.24)	2.03 (1.13–3.67)	1.98 (1.02–3.83)	0.023	
High	1.00	0.85 (0.37–1.96)	1.25 (0.54–2.91)	1.38 (0.57–3.34)	0.182	
**Income**						0.960
Low	1.00	0.96 (0.66–1.40)	1.15 (0.73–1.80)	1.11 (0.63–1.96)	0.583	
Medium	1.00	0.81 (0.53–1.23)	0.99 (0.63–1.57)	1.08 (0.63–1.86)	0.513	
High	1.00	1.02 (0.58–1.79)	1.75 (0.99–3.11)	1.98 (1.07–3.67)	0.003	
**Urbanization**						0.107
Low	1.00	0.71 (0.45–1.12)	0.85 (0.47–1.53)	1.06 (0.51–2.21)	0.855	
Medium	1.00	0.98 (0.65–1.46)	1.54 (0.98–2.41)	2.14 (1.26–3.65)	0.002	
High	1.00	0.82 (0.50–1.34)	1.01 (0.62–1.67)	1.04 (0.61–1.77)	0.444	
**Smoking**						0.146
Non smoker	1.00	1.16 (0.86–1.56)	1.47 (1.06–2.06)	1.61 (1.09–2.37)	0.009	
Current smokers	1.00	0.54 (0.33–0.90)	0.86 (0.51–1.44)	1.02 (0.57–1.82)	0.358	
**Hypertension**						0.746
No	1.00	0.93 (0.66–1.30)	1.28 (0.89–1.85)	1.50 (0.99–2.27)	0.016	
Yes	1.00	0.94 (0.64–1.38)	1.26 (0.83–1.92)	1.30 (0.80–2.09)	0.148	
**Overweight**						0.769
No	1.00	0.85 (0.60–1.20)	1.08 (0.73–1.58)	1.05 (0.67–1.64)	0.547	
Yes	1.00	1.00 (0.68–1.48)	1.50 (0.98–2.28)	1.80 (1.12–2.89)	0.003	

Values are odds ratios with a 95% CI from mixed-effect logistic regression models. The model was adjusted for age, gender, energy intake, education, income, urbanization, smoking, alcohol drinking, and physical activity, dietary patterns, BMI, and hypertension. Q, quartile. Stratification variables were not adjusted in the corresponding models.

**Table 4:** Subgroup analyses of the association between the quartiles of plant methionine intake and diabetes.

	Q1	Q2	Q3	Q4	*p* _trend_	*p* _interaction_
**Age (in years)**						0.776
<60 years	1.00	1.00 (0.75–1.32)	0.96 (0.70–1.31)	1.13 (0.79–1.62)	0.614	
≥60 years	1.00	0.86 (0.63–1.17)	1.08 (0.74–1.57)	1.10 (0.68–1.80)	0.653	
**Sex**						0.725
Men	1.00	1.08 (0.77–1.53)	1.17 (0.81–1.68)	1.30 (0.86–1.96)	0.203	
Women	1.00	0.94 (0.72–1.22)	1.06 (0.76–1.46)	1.31 (0.87–1.99)	0.299	
**Education**						0.576
Low	1.00	0.85 (0.64–1.15)	1.18 (0.85–1.63)	1.32 (0.89–1.95)	0.106	
Medium	1.00	1.14 (0.76–1.72)	0.91 (0.57–1.46)	0.98 (0.56–1.72)	0.733	
High	1.00	1.01 (0.66–1.55)	0.96 (0.57–1.62)	1.37 (0.71–2.63)	0.538	
**Income**						0.155
Low	1.00	0.95 (0.63–1.41)	1.38 (0.91–2.10)	1.42 (0.87–2.33)	0.076	
Medium	1.00	1.28 (0.88–1.85)	1.02 (0.67–1.54)	1.04 (0.64–1.70)	0.910	
High	1.00	0.94 (0.68–1.30)	1.06 (0.71–1.56)	1.43 (0.88–2.33)	0.231	
**Urbanization**						0.064
Low	1.00	1.28 (0.66–2.49)	1.52 (0.80–2.89)	2.06 (1.05–4.05)	0.023	
Medium	1.00	1.07 (0.72–1.59)	1.08 (0.70–1.66)	1.00 (0.61–1.65)	0.974	
High	1.00	0.96 (0.73–1.26)	1.07 (0.76–1.51)	1.25 (0.79–1.97)	0.407	
**Smoking**						0.376
Non smoker	1.00	0.95 (0.75–1.21)	1.01 (0.76–1.35)	1.28 (0.90–1.81)	0.258	
Current smokers	1.00	1.08 (0.70–1.65)	1.21 (0.77–1.89)	1.22 (0.73–2.04)	0.391	
**Hypertension**						0.666
No	1.00	1.01 (0.77–1.34)	1.10 (0.81–1.50)	1.35 (0.93–1.95)	0.131	
Yes	1.00	0.95 (0.71–1.28)	1.00 (0.71–1.41)	0.99 (0.65–1.52)	1.000	
**Overweight**						0.487
No	1.00	0.93 (0.70–1.24)	1.16 (0.83–1.60)	1.17 (0.79–1.75)	0.304	
Yes	1.00	1.02 (0.76–1.37)	0.96 (0.68–1.35)	1.21 (0.80–1.81)	0.529	

Values are odds ratios with a 95% CI from mixed-effect logistic regression models. The model was adjusted for age, gender, energy intake, education, income, urbanization, smoking, alcohol drinking, and physical activity, dietary patterns, BMI, and hypertension. Q, quartile. Stratification variables were not adjusted in the corresponding models.

## Data Availability

The datasets are available in the CHNS repository at https://www.cpc.unc.edu/projects/china.
